# Mediating Effect of Sleep Quality on the Association Between Psychological Stress and Acne Vulgaris in Chinese College Students: A Cross-Sectional Study

**DOI:** 10.3390/healthcare14111481

**Published:** 2026-05-27

**Authors:** Mei-Hua Chen, Ling Ma, Liu-Qing Chen, Li Qin

**Affiliations:** 1 Department of Dermatology, Traditional Chinese and Western Medicine Hospital of Wuhan, Tongji Medical College, Huazhong University of Science and Technology, Wuhan 430022, China; meizixxs@163.com (M.-H.C.); maling555@163.com (L.M.); chlq35@126.com (L.-Q.C.); 2 Department of Dermatology, Wuhan No. 1 Hospital, No. 215 Zhongshan Avenue, Qiaokou District, Wuhan 430022, China; 3 Hubei Province & Key Laboratory of Skin Infection and Immunity, Wuhan 430022, China

**Keywords:** acne vulgaris, college students, psychological stress, sleep quality, mediation effect

## Abstract

**Objective**: To identify modifiable factors associated with acne vulgaris in college students and further delineate the potential mechanistic pathway underlying the effect of psychological stress on acne development. **Methods**: We conducted a cross-sectional study with a questionnaire-based survey among 819 college students, collecting data on demographic characteristics, lifestyle behaviors, sleep quality, psychological stress, and acne prevalence. Descriptive statistics and univariate analyses were used to compare baseline characteristics between groups, and stepwise adjusted binary logistic regression models were constructed to identify independent risk factors for acne. The Bootstrap method was applied for mediation effect analysis. Subgroup analyses, interaction tests, sensitivity analyses, and robustness analyses were also performed. **Results**: The overall prevalence of acne in the study cohort was 33.33%. Multivariable regression analysis revealed that total stress score (OR = 1.187, *p* < 0.001), poor sleep quality (OR = 1.109, *p* = 0.010), excessive screen time (OR = 1.107, *p* = 0.021), intake of sugary drinks (OR = 1.561, *p* = 0.027), spicy diet (OR = 1.739, *p* = 0.003), smoking (OR = 1.809, *p* = 0.031), and use of skincare products (OR = 2.004, *p* = 0.001) were independent risk factors for acne, while outdoor activities were a protective factor (OR = 0.676, *p* = 0.048). Mediation effect analysis demonstrated that psychological stress exerted not only a direct effect on acne development, but also an indirect effect to increase disease risk via impairing sleep quality. This indirect effect was statistically significant (95% CI: 0.000–0.013, *p* < 0.05), accounting for approximately 20.67% of the total effect. The sensitivity analysis revealed that the E-values of all significant variables were greater than 1.4. The robustness test indicated that the results remained unchanged after excluding students majoring in medicine and extreme values. Furthermore, LASSO variable screening and Bootstrap internal validation further verified the stability of the model. **Conclusions**: Although as a cross-sectional study, this work cannot establish causal relationships, but we found that psychological stress and sleep disturbance are key modifiable risk factors for acne, and the impact of stress on acne is partially mediated by the impairment of sleep quality.

## 1. Introduction

Acne vulgaris is a chronic inflammatory skin disease of the pilosebaceous unit, with a predilection for adolescents and young adults [1,2]. Epidemiological data indicate that more than 85% of adolescents experience acne of varying severity, and the condition maintains a high prevalence among college students globally [3,4]. Beyond cutaneous damage, acne is closely linked to adverse psychological outcomes, including anxiety and depression, and exerts a substantial negative impact on social confidence and health-related quality of life in affected individuals [5,6,7]. Therefore, identifying modifiable risk factors for acne in college students is of critical public health importance for the development of targeted prevention strategies.

The pathogenesis of acne is driven by the combined effects of multiple factors, including genetic predisposition, endocrine dysregulation, environmental exposures, and lifestyle behaviors. Existing research has established that aberrant androgen levels are the core intrinsic driver of excessive sebaceous gland secretion [8]. In addition, modifiable lifestyle behaviors play a pivotal role in the onset and progression of acne. Previous studies have linked lifestyle factors such as a high-glycemic diet, physical inactivity, and excessive screen time to an elevated risk of acne development [9,10,11].

However, lifestyle factors alone do not fully explain the pathogenesis of acne. In recent years, a growing body of research has highlighted the role of psychological factors in acne development. Modern psychophysiological studies have demonstrated that psychological stress activates the hypothalamic–pituitary–adrenal (HPA) axis, triggering hypothalamic secretion of corticotropin-releasing hormone (CRH), which in turn stimulates pituitary release of adrenocorticotropic hormone (ACTH) and subsequent adrenal cortisol secretion. Cortisol not only acts directly on sebaceous glands to drive excess sebum production, but also targets keratinocytes and cutaneous immune cells to promote the release of pro-inflammatory cytokines, including tumor necrosis factor-α (TNF-α), interleukin (IL)-6, and IL-8, thereby exacerbating perifollicular inflammatory responses [12,13].

We hypothesize that the impact of stress on acne is not entirely direct, but may be indirectly mediated through a series of intermediate variables. Sleep quality is among the most plausible mediating factors. Individuals exposed to chronic stress frequently experience sleep onset difficulties, sleep maintenance disorders, and fragmented sleep, leading to a marked reduction in sleep quality [14,15]. Concurrently, accumulating evidence indicates that sleep itself is a key regulator of cutaneous homeostasis. Sleep deprivation impairs skin barrier function, resulting in increased transepidermal water loss (TEWL); meanwhile, it significantly elevates circulating inflammatory cytokine levels, exacerbating systemic inflammation. Furthermore, sleep disturbance disrupts the circadian rhythm of cortisol secretion, leading to abnormally elevated nocturnal cortisol levels that further drive sebum production [16]. On this basis, we postulate that stress may first impair sleep in college students, with subsequent sleep disturbance triggering acne onset. In other words, sleep quality may serve as a critical intermediary bridge between stress and acne.

In addition, screen time may function as another potential mediating variable. According to behavioral psychology theory, individuals facing stress often adopt avoidance coping strategies, such as excessive engagement with smartphones and online games, to temporarily escape real-world stressors [17]. This suggests that students experiencing higher levels of stress may have longer daily screen time. Moreover, excessive screen time has been previously associated with acne, as blue light exposure, prolonged sedentary behavior, and the consequent reduction in outdoor activity may all exacerbate cutaneous pathology. We therefore hypothesize that a “stress → screen time → acne” pathway may represent an additional mechanistic link between stress and acne.

Recent studies have further confirmed the independent associations between these factors and acne. For example, Zhu et al. reported the global burden of acne in young adults, highlighting the urgent need for identifying modifiable risk factors [18]; He et al. found that beverage consumption was significantly associated with increased acne risk in Chinese adults [19]; and Celik et al. revealed the bidirectional relationship between acne treatment and sleep quality, further supporting the interaction between these two factors [20]. However, no studies have systematically investigated these two potential mediating pathways. Most existing research has analyzed the isolated associations between stress, sleep, or screen time and acne, with few studies integrating these factors into a unified analytical framework to delineate their sequential relationships.

Based on the above theoretical background, we proposed three primary hypotheses:

1. Higher level of psychological stress is independently associated with increased risk of acne vulgaris in college students, after adjusting for demographic and lifestyle covariates.
2. Sleep quality mediates the association between psychological stress and acne, such that stress impairs sleep quality, which in turn increases acne risk.
3. Screen time mediates the association between psychological stress and acne, such that stress increases daily screen time, which in turn increases acne risk.

In this study, we defined acne prevalence as the primary outcome variable, psychological stress as the main exposure variable, sleep quality and daily screen time as the potential mediating variables, and demographic characteristics and lifestyle behaviors as covariates. This analytical framework allows us to systematically disentangle the direct and indirect effects of stress on acne.

As a cross-sectional study, this work cannot establish causal relationships, but it aims to explore the potential mechanistic pathways based on theoretical temporal ordering. Specifically, based on a large cross-sectional survey of Chinese college students, this study aims to: (1) systematically evaluate the prevalence of acne and its associated influencing factors in college students; (2) quantify the contribution of variables from different domains to acne risk using stepwise adjusted regression models; and (3) examine the two potential mediating pathways of “stress → sleep quality → acne” and “stress → screen time → acne” via formal mediation effect analysis. This work seeks to elucidate the mechanistic basis underlying the stress-acne association and to provide a theoretical foundation for the comprehensive prevention and control of acne in the college student population.

## 2. Materials and Methods

### 2.1. Study Population

This cross-sectional study recruited 819 college students from two comprehensive universities in Wuhan, China, via convenience sampling. Eligible participants were full-time undergraduate students enrolled at the target institutions, with no restrictions on age, gender, grade, or major. Participants were excluded if they had severe systemic disease, other chronic inflammatory skin disorders, or a history of long-term systemic glucocorticoid or anti-androgen medication use. All participants provided written informed consent prior to study enrollment. All procedures were conducted in strict adherence to the tenets of the Declaration of Helsinki. The Ethics Committee of Wuhan First Hospital has formally reviewed and confirmed that this study is exempt from ethical review.

We adopted convenience sampling to recruit participants from two universities in Wuhan. While this sampling method may introduce potential selection bias, we included participants from different grades, majors, and gender groups to maximize the representativeness of the sample. Furthermore, we conducted robustness analyses to verify the stability of our results, which helps to mitigate the impact of potential selection bias.

### 2.2. Data Collection and Measurement Tools

Data were collected using a structured, self-administered, anonymous questionnaire, which included assessments of demographic characteristics, lifestyle behaviors, sleep quality, and psychological stress levels. The survey was distributed online via the Wenjuanxing platform (https://www.wjx.cn/), a widely used and validated online survey tool in China.

### 2.3. Study Variables

#### 2.3.1. Outcome Variable: Acne Prevalence

Acne prevalence was assessed using a validated self-reported question: “Have you been troubled by acne (pimples) in the past week?” Participants who answered “Yes” were defined as acne cases, while those who answered “No” were defined as controls. We explained the symptoms and diagnosis of acne to each respondent in the questionnaire. However, we acknowledge that this measure cannot assess the severity of acne, which is a limitation of the current study.

#### 2.3.2. Exposure Variable: Psychological Stress Assessment

The Chinese College Student Stress Inventory (C-CSI) was used to evaluate participants’ perceived stress levels. This validated scale consists of 18 items rated on a 5-point Likert scale (1 = “completely disagree” to 5 = “completely agree”), encompassing three dimensions: academic stress, economic stress, and social stress (6 items per dimension). Higher total and dimension-specific scores indicate greater levels of perceived stress. The Cronbach’s α coefficient for the overall scale is 0.90, with coefficients of 0.91, 0.90, and 0.88 for the academic stress, economic stress, and social stress subscales, respectively, demonstrating excellent reliability and validity for stress assessment in Chinese undergraduate students.

#### 2.3.3. Mediating Variables

(1) Sleep Quality: The Pittsburgh Sleep Quality Index (PSQI) was used to assess participants’ sleep quality over the preceding month [21]. The scale comprises 19 self-rated items grouped into 7 dimensions: subjective sleep quality, sleep latency, sleep duration, habitual sleep efficiency, sleep disturbances, use of sleep medication, and daytime dysfunction. Each dimension is scored from 0 to 3, with a total global score ranging from 0 to 21 (calculated as the sum of the 7 dimension scores). Higher total scores indicate poorer sleep quality. In the present study, the Cronbach’s α coefficient of the PSQI was 0.91, indicating good internal consistency reliability in the study cohort.
(2) Screen Time: Assessed as the average daily duration of electronic device use (in hours).

#### 2.3.4. Covariates: Demographic and Lifestyle Factors

Demographic data collected included age, gender, grade, and major. Lifestyle variables included daily screen time, frequency of outdoor physical activity, dietary intake (sugary beverages, fried foods, fresh vegetables, and fruits), smoking status, alcohol consumption, and skincare product use (defined as the average daily frequency within the past month). All lifestyle variables were assessed using validated categorical items consistent with prior public health surveys targeting Chinese college students.

### 2.4. Statistical Analysis

All statistical analyses were conducted using Python version 3.14.3 (Beaverton, U.S.). A two-sided *p*-value < 0.05 was considered statistically significant for all tests.

#### 2.4.1. Descriptive and Univariate Statistical Methods

For continuous variables, between-group differences were compared using the independent samples *t*-test (for normally distributed data) or the Mann–Whitney U test (for non-normally distributed data). For categorical variables, the chi-square (χ^2^) test was used to compare differences between groups. Results were presented in Table 1 in accordance with standard SCI reporting guidelines.

#### 2.4.2. Multivariable Logistic Regression Analysis

Four stepwise adjusted models were constructed to evaluate the associations between the variables of interest and acne. Model 1 included only lifestyle variables; Model 2 additionally adjusted for demographic variables; Model 3 further incorporated sleep quality; and Model 4 (the final fully adjusted model) included the three stress dimensions. Odds ratios (ORs), 95% confidence intervals (CIs), and *p*-values were calculated for each variable in all models. The discriminatory performance of each model was evaluated using the area under the receiver operating characteristic (ROC) curve (AUC).

#### 2.4.3. Mediation Effect Analysis Procedures

The Bootstrap method with 5000 resamples was used to test the statistical significance of mediation effects. A mediation effect was considered statistically significant if the 95% CI for the indirect effect did not contain 0.

#### 2.4.4. Subgroup Analysis Procedures

Subgroups were divided based on gender, grade (lower/upper), major (medical/non-medical), outdoor activities, and origin. The differences in the effect of stress on acne among different subgroups were analyzed, and interaction *p* values were calculated to test heterogeneity.

#### 2.4.5. Interaction Test Procedures

The effects of four interaction terms, namely gender × stress, sleep × stress, gender × sleep, and stress × sugar-sweetened beverages, were tested.

#### 2.4.6. Sensitivity Analysis Procedures

Calculate the E-value for significant variables in the main model to assess the impact of unmeasured confounding; simultaneously, standardize continuous variables and refit the model to compare the consistency of the results.

#### 2.4.7. Robustness Test Procedures

After excluding students majoring in medicine and extreme values of continuous variables, the model was refitted [22]. LASSO regression was used to screen variables and verify the stability of the model. The main model underwent 500 bootstrap resamplings, and the 95% confidence interval (CI) of the Area Under the Curve (AUC) was calculated to assess the stability of the model’s discriminatory power.

## 3. Results

### 3.1. Descriptive and Univariate Analyses

A total of 819 valid questionnaires were included in the final analysis. Of these, 273 participants reported acne symptoms in the prior week, yielding an overall acne prevalence of 33.33%. Statistically significant differences between the acne and control groups were observed for screen time, sleep quality, sugary beverage intake, outdoor activity, fried food intake, vegetable and fruit intake, smoking status, alcohol consumption, skincare product use, academic stress, and social stress (all *p* < 0.05) (Table 1).

### 3.2. Multivariable Stepwise Regression Analysis Results

**Figure 1:**
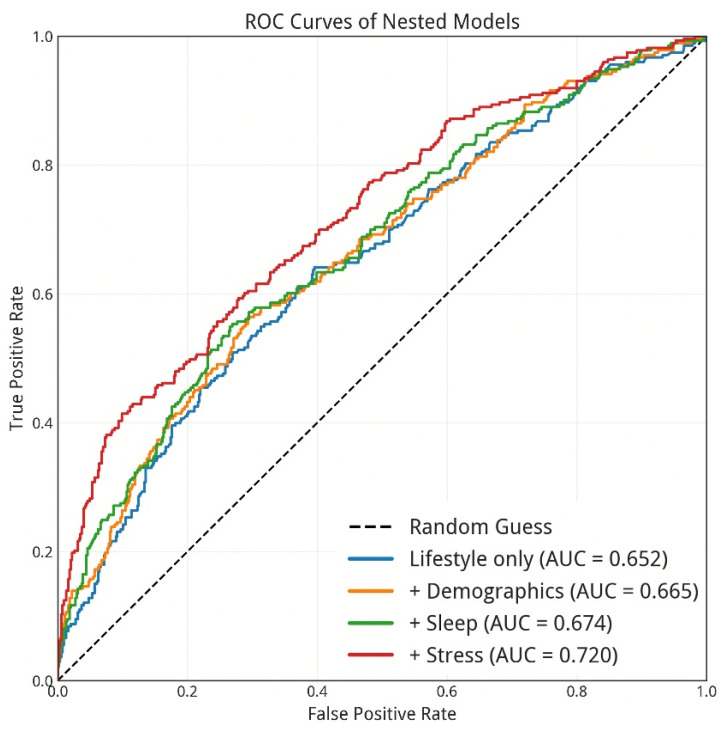
Multivariable stepwise regression analysis.

**Figure 2:**
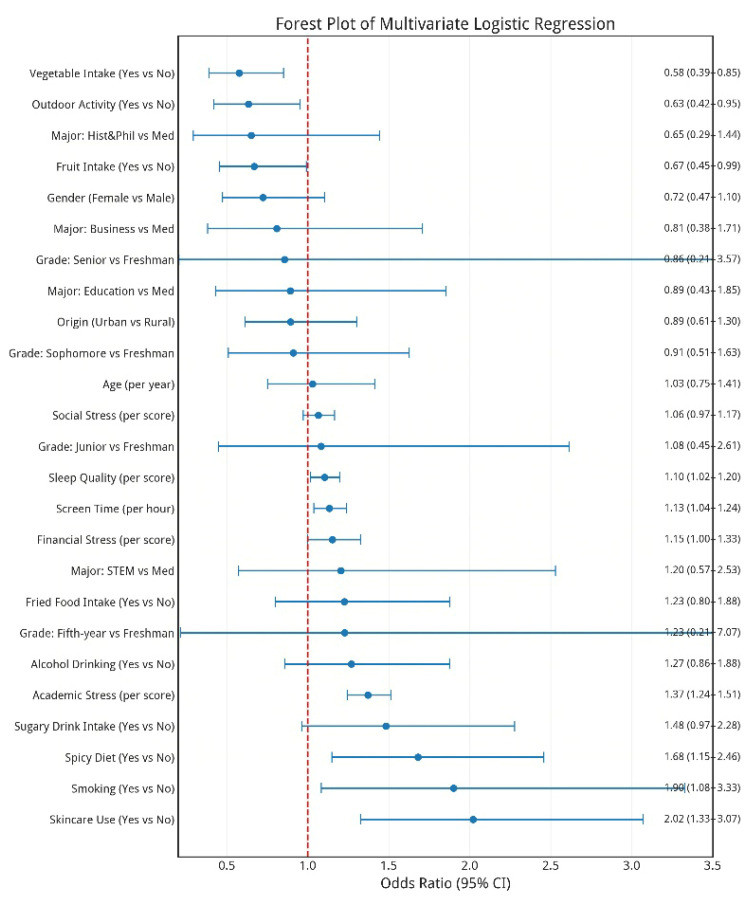
Forest plot of multivariable logistic regression. Comparison of receiver operating characteristic (ROC) curves for the prediction of acne occurrence in college students, using models with different variable sets. Forest plot of adjusted odds ratios (ORs) and 95% confidence intervals (CIs) for acne-associated risk factors from the fully adjusted multivariate logistic regression model. Variables are ordered by the magnitude of their effect sizes. The vertical dashed line at OR = 1 indicates the null effect. Variables with 95% CIs not crossing the null line represent statistically significant independent associations after adjustment for all other covariates in the model.

We constructed a 4-layer nested logistic regression model, gradually incorporating variables from different dimensions to assess their contribution to the model’s discriminatory power. The results showed that the AUC of the basic model incorporating only lifestyle variables was 0.652. After adding demographic variables, the AUC of the model increased to 0.665. Further incorporating variables related to sleep quality raised the AUC to 0.674. Finally, after adding variables related to psychological stress, the AUC of the model reached 0.720, indicating that psychological stress variables significantly improved the model’s discriminatory ability for acne. The final multifactor regression results showed that after adjusting for all confounding variables, total stress score, sleep quality, screen time, intake of sugary drinks, spicy diet preference, smoking, and use of skincare products were all independent risk factors for acne, with corresponding OR values of 1.187, 1.109, 1.107, 1.561, 1.739, 1.809, and 2.004, respectively. When examining the three sub-domains of stress separately, only academic stress showed a statistically significant association with acne (OR = 1.37, 95% CI: 1.24–1.51, *p* < 0.001), while social stress (OR = 1.06, 95% CI: 0.97–1.17, *p* = 0.172) and economic stress (OR = 1.15, 95% CI: 1.00–1.33, *p* = 0.052) had weaker and non-significant effects, indicating that academic stress is the primary driver of the observed stress-acne association. Outdoor activities were a protective factor for acne, with an OR value of 0.676. All the above variables had *p* values less than 0.05, indicating statistical significance (Table 2 and Figure 1). The forest plot further visualizes the independent effects of each variable (Figure 2). The results indicate that the inclusion of psychological stress variables significantly improved the model’s discriminatory power. Although the incremental improvement in AUC was moderate, this result demonstrates that psychological stress provides independent predictive value for acne risk, beyond the traditional lifestyle and demographic factors.

### 3.3. Mediation Effect Analysis

**Figure 3:**
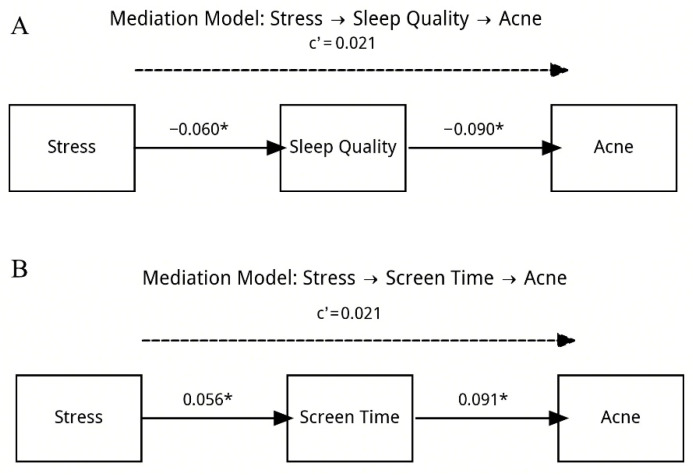
Mediation effect path diagram. Mediation models examining the indirect effects of stress on acne via sleep quality and screen time. (**A**) The mediation model of stress → sleep quality → acne. (**B**) The mediation model of stress → screen time → acne. Path coefficients were estimated using linear regression for the mediator model and logistic regression for the binary acne outcome, with 5000 bootstrap resamples employed to calculate the 95% confidence intervals of the indirect effects. Values adjacent to the arrows indicate the regression coefficients. An asterisk (*) denotes that the path coefficient is statistically significant at the *p* < 0.05 level. The dashed line represents the direct effect of stress on acne, while the solid lines represent the indirect paths through the respective mediators. This analysis was conducted on 819 valid participants.

Stress exerted a significant negative effect on sleep quality (path a coefficient = −0.060, *p* < 0.05). Specifically, each 1-point increment in stress level was associated with an average 0.06-point reduction in sleep quality score. Sleep quality showed a significant negative association with acne occurrence (path b coefficient = −0.090, *p* < 0.05): for each 1-point decrease in sleep quality score, the log-odds of acne occurrence increased by 0.09, indicating that poorer sleep quality correlates with an elevated risk of acne development. The 95% confidence interval (CI) for the indirect effect of stress on acne mediated by sleep quality was [0.000, 0.013], which did not contain zero, confirming the statistically significant mediating effect of sleep quality (Figure 3A). Collectively, these findings demonstrate that stress increases the risk of acne occurrence by impairing sleep quality, with this mediating pathway accounting for 20.67% of the total effect of stress on acne.

In contrast, although stress was positively associated with screen time, and screen time was in turn linked to an increased risk of acne, this entire mediating pathway did not reach statistical significance; thus, the presence of this mediating effect could not be statistically verified (Figure 3B).

### 3.4. Subgroup Analysis and Interaction Tests

**Figure 4:**
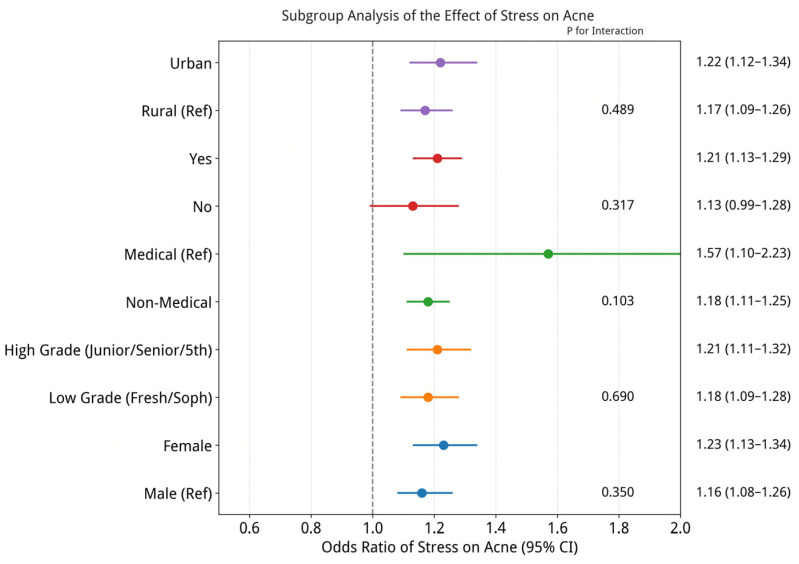
Forest plot of subgroup analysis for the effect of total stress on acne. This figure shows the odds ratios (ORs) and 95% confidence intervals (CIs) of the effect of total stress on acne across different subgroups, including gender, grade (lower grade: freshman/sophomore; higher grade: junior/senior/fifth-year), major (medical vs. non-medical), outdoor activity, and student origin (urban vs. rural). Each OR represents the change in odds of acne per 1-point increase in total stress score within that subgroup. All subgroup models were adjusted for age to control for potential confounding. *p* for interaction was calculated to test the heterogeneity of the effect across subgroups. The vertical dashed line represents the reference line of OR = 1, indicating no effect of stress on acne.

To explore whether the effect of stress on acne varies across different populations, we conducted a subgroup analysis, grouping participants based on gender, grade, major, outdoor activities, and place of origin. The results indicated that in all subgroups, the effect of stress on acne was positive, consistent with the findings of the main model. All interaction *p* values were greater than 0.05: gender interaction *p* = 0.244, grade interaction *p* = 0.312, major interaction *p* = 0.427, outdoor activities interaction *p* = 0.189, and place of origin interaction *p* = 0.513 (Table 3 and Figure 4). This suggests that the effect of stress on acne is consistently present across different subgroups of college students, with no significant heterogeneity, indicating that our results have good generalizability and are applicable to college students with different characteristics.

**Figure 5:**
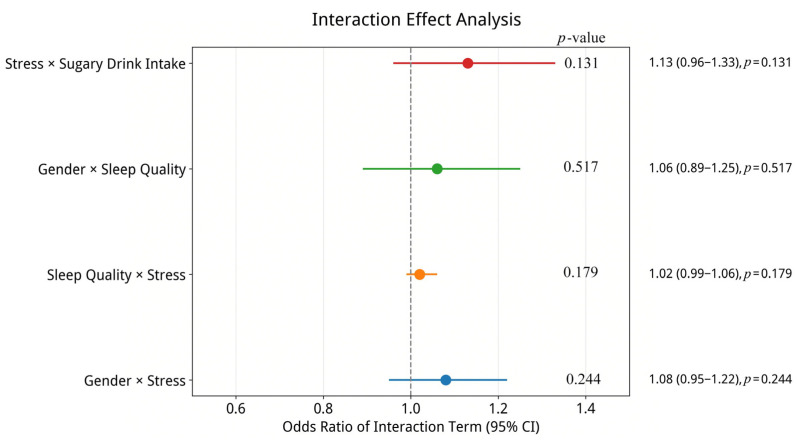
Forest plot of interaction effects between variables on acne. This figure shows the odds ratios (ORs) and 95% confidence intervals (CIs) of the four pre-specified interaction terms, including gender × total stress, sleep quality × total stress, gender × sleep quality, and total stress × sugary beverage intake. The model was adjusted for all covariates in the full model, including age, gender, total stress, sleep quality, screen time, sugary beverage intake, outdoor activity, spicy diet, smoking, and skincare product use. The vertical dashed line represents the reference line of OR = 1, indicating no interaction effect.

We further examined four potential interactions, and the results indicated that none of the interaction terms reached statistical significance (*p* > 0.05) (Table 4 and Figure 5). This suggests that there is no significant interaction among the variables we studied, and the effects of the variables are independent, without synergistic or antagonistic interactions.

### 3.5. Sensitivity Analyses

To verify the robustness of the results, we conducted a sensitivity analysis. We calculated the E-value for all significant variables in the main model. The results showed that the E-value for using skincare products was the highest, at 3.42, indicating that to refute this association, unmeasured confounding would need to simultaneously affect both the exposure and the hazard ratio of the outcome to the same extent. The E-value for smoking was 3.02, for eating spicy food was 2.87, for drinking sugary beverages was 2.50, for outdoor activities was 2.32, for stress scores was 1.66, for sleep quality was 1.46, and for screen time was 1.45. All E-values were greater than 1.4, indicating that unmeasured confounding factors are unlikely to refute the observed association, and the results are very robust (Table 5).

After standardizing all continuous variables, we refitted the model. The results showed that the effect direction and significance of all variables were completely consistent with the original model, indicating that the results would not change due to changes in the scale of the variables, and the reliability of the results was high.

### 3.6. Robustness Test

Considering the possibility of self-medication among medical students, we excluded this part of the sample and refitted the model. The results showed that the effect direction and significance of all variables were completely consistent with the original model. We excluded extreme values outside 3σ in continuous variables and refitted the model, and the results were also completely consistent with the original model, indicating that the results were not affected by extreme values. We used LASSO regression for variable selection, and the results showed that the core variables selected were basically consistent with those in our original main model, indicating that our variable selection was stable and there was no overfitting issue. We conducted 500 bootstrap self-sampling on the main model, and the results showed that the mean AUC of the model was 0.698, with a 95% CI of 0.661–0.735 (Table 6). The confidence interval was very narrow, indicating that the model’s discriminatory power was very stable and would not undergo significant changes due to sample fluctuations.

## 4. Discussion

In this large cross-sectional study of 819 college students, we provide the first systematic evidence delineating the mediating mechanisms underlying the association between psychological stress and acne vulgaris. We found that the prevalence of acne among Chinese college students was 33.33%, which is consistent with prior epidemiological studies, and reaffirms that acne is a highly prevalent health issue in this population [22,23]. More importantly, we not only identified psychological stress, sleep quality, screen time, dietary factors, smoking, and use of skin care products, but also uncovered a key mechanistic finding: over a quarter of the adverse effects of psychological stress on acne are indirectly mediated through the impairment of sleep quality. While our cross-sectional design cannot establish definitive causal relationships, these findings provide strong preliminary evidence for a sequential pathway linking stress to sleep disturbance to acne, which is supported by robust biological mechanisms. This discovery provides a novel perspective for understanding the pathogenesis of psychosomatic dermatological disorders and identifies new actionable targets for clinical and public health interventions.

The most pivotal finding of this study is the significant mediating effect of sleep quality in the association between stress and acne. This result robustly supports our a priori hypothesis that stress does not act solely on the skin directly via the HPA axis, but also indirectly exacerbates cutaneous inflammation through sleep disturbance as a critical intermediate link [16,20]. From a physiological perspective, this sequential pathway is biologically plausible. First, chronic psychological stress—particularly the academic stress faced by college students—places the body in a persistent state of physiological arousal. This state disrupts hypothalamic circadian clock regulation, delays melatonin secretion, and elevates nocturnal cortisol levels, ultimately leading to sleep onset difficulties, sleep maintenance disorders, and fragmented sleep. Impaired sleep quality, in turn, exerts a cascade of deleterious effects on cutaneous homeostasis [24,25].

Among the different dimensions of stress, we found that academic stress was the strongest predictor of acne, while the effects of social and economic stress were relatively weaker. This finding is highly congruent with the unique characteristics of the college student population [26]. Although the incremental improvement in AUC was moderate, this result demonstrates that psychological stress provides independent predictive value for acne risk, beyond the traditional lifestyle and demographic factors. In the context of multifactorial chronic diseases like acne, even moderate improvements in predictive performance can have important clinical and public health implications, particularly when they identify novel modifiable risk factors and mechanistic pathways.

Notably, we did not detect a significant mediating effect of screen time in the stress-acne association. While screen time was correlated with acne in univariate analyses, the indirect effect of stress on acne via screen time was not statistically significant in the mediation model. This may indicate that the impact of screen time on acne is independent, rather than mediating, i.e., it acts as a separate risk factor rather than an intermediate step in the stress-acne pathway. Alternatively, the sample size of the current study may have been insufficient to detect a small indirect effect, and future studies should further distinguish between different types of screen time (e.g., academic vs. recreational use) to validate this finding [27,28]. In addition, a potential reason is that we measured total daily screen time without differentiating between academic and recreational use. Academic screen time increased by stress may not have the same impact on acne as recreational screen time (e.g., late-night blue light exposure). Future studies need to differentiate between different types of screen time to further explore this pathway.

In terms of dietary factors, this study found that intake of sugary beverages and a spicy diet are risk factors for acne, while outdoor activities are protective factors. Previous studies have found that a high-sugar diet can induce acne by increasing the level of insulin-like growth factor-1 (IGF-1), promoting sebum secretion, and exacerbating inflammatory reactions [19,29]. The results of this study further validate this point. Regarding a spicy diet, previous studies have been controversial, but this study found that it is a risk factor for acne. This may be because spicy foods can exacerbate skin inflammation, dilate blood vessels, and thus aggravate acne symptoms. In addition, we found that outdoor activities are protective factors, possibly because they can increase vitamin D synthesis, which can regulate immune function and reduce inflammatory reactions. At the same time, outdoor activities also imply more exercise, improving metabolism and thus reducing the risk of acne [18,30].

An important advantage of this study is that we conducted a comprehensive subgroup analysis and interaction tests. The results showed that our core effects were consistently present in different subgroups of the population, with no significant heterogeneity. At the same time, no significant interactions were found, indicating that our results have good universality. Regardless of gender, grade level, major (medical or non-medical), or location (urban or rural), the effects of these risk factors are consistent. This also suggests that our intervention measures can be applied to all university student groups, without the need for special adjustments for different subgroups.

In addition, we conducted comprehensive sensitivity and robustness tests, all of which verified the reliability of our results. We noted that while all E-values are greater than 1.4, several are close to 1.5, which means a modest unmeasured confounder could potentially alter our results, highlighting the need for more confounders in future studies. The exclusion of medical students, the exclusion of extreme values, LASSO variable screening, and Bootstrap internal validation further verified the robustness of our results, indicating that our findings are not altered by changes in samples or variable scales, and the model exhibits high stability.

This study has several limitations that should be acknowledged. First, the cross-sectional study design precludes the establishment of definitive causal relationships between variables. For example, it is possible that acne itself may induce psychological stress and sleep disturbance. While the direction of our mediation analysis is grounded in established biological and psychological theory, future prospective cohort studies are needed to validate the causal direction of the observed associations. Second, acne status was determined via self-report rather than clinical diagnosis by a dermatologist, which may introduce some degree of misclassification bias. However, self-reported acne assessment is a widely used and validated method in large-scale epidemiological surveys. Finally, we did not collect detailed data on BMI, hormonal status, or family acne history, which may act as potential unmeasured confounding factors.

## 5. Conclusions

Overall, this study systematically reveals the multiple influencing factors of acne in college students, including psychological stress, sleep quality, screen time, dietary factors, smoking, etc. The results are robust and reliable, with good universality. While our cross-sectional design precludes definitive causal conclusions, the observed pathways are biologically plausible and consistent with established psychophysiological mechanisms. Our research suggests that comprehensive intervention measures should be taken for acne prevention among college students, including alleviating psychological stress, improving sleep health, reducing screen time, advocating for a healthy diet, reducing the intake of high-sugar and spicy foods, increasing outdoor activities, and quitting smoking. These measures may effectively reduce the risk of acne occurrence in college students and improve their skin health and quality of life.

## Figures and Tables

**Table 1:** Baseline characteristics of the participants (for categorical variables, data is represented as n (%)).

Variable	Acne Group (n = 273)	Non-Acne Group (n = 546)	U/χ^2^-Value	*p*-Value
Age	20.0 (19.0–21.0)	20.0 (19.0–21.0)	73,924.50	0.847
Gender			0.52	0.470
Female	125 (45.8)	234 (42.9)		
Male	148 (54.2)	312 (57.1)		
Place of origin			0.05	0.820
Rural	166 (60.8)	326 (59.7)		
Urban	107 (39.2)	220 (40.3)		
Screen time (h/day)	4.1 (2.9–5.4)	4.0 (2.7–5.2)	80,760.50	0.051
Sleep quality	5.0 (4.0–7.0)	5.0 (4.0–7.0)	68,086.00	0.041 *
Grade			0.96	0.916
Grade 1	68 (24.9)	133 (24.4)		
Grade 2	74 (27.1)	156 (28.6)		
Grade 3	85 (31.1)	160 (29.3)		
Grade 4	30 (11.0)	69 (12.6)		
Grade 5	16 (5.9)	28 (5.1)		
Major			6.29	0.098
Education	85 (31.1)	174 (31.9)		
History & Philosophy	32 (11.7)	92 (16.8)		
Medicine	15 (5.5)	30 (5.5)		
Science & Engineering	77 (28.2)	119 (21.8)		
Sugary beverage intake (time/day)			5.01	0.025 *
≥1	60 (22.0)	84 (15.4)		
<1	213 (78.0)	462 (84.6)		
Outdoor activity (time/day)			4.33	0.037 *
≥1	212 (77.7)	458 (83.9)		
<1	61 (22.3)	88 (16.1)		
Fried food intake (time/day)			4.11	0.043 *
≥1	60 (22.0)	87 (15.9)		
<1	213 (78.0)	459 (84.1)		
Vegetable intake (time/day)			5.57	0.018 *
≥1	204 (74.7)	448 (82.1)		
<1	69 (25.3)	98 (17.9)		
Fruit intake (time/day)			5.73	0.017 *
≥1	206 (75.5)	452 (82.8)		
<1	67 (24.5)	94 (17.2)		
Smoking (time/day)			4.61	0.032 *
≥1	30 (11.0)	35 (6.4)		
<1	243 (89.0)	511 (93.6)		
Alcohol consumption (time/day)			3.56	0.059
≥1	65 (23.8)	98 (17.9)		
<1	208 (76.2)	448 (82.1)		
Spicy preference			9.09	0.003 **
Bland	192 (70.3)	437 (80.0)		
Spicy	81 (29.7)	109 (20.0)		
Skincare product use			8.39	0.004 **
No	125 (45.8)	310 (56.8)		
Yes	148 (54.2)	236 (43.2)		
Total stress score	24.0 (23.0–26.0)	24.0 (22.0–26.0)	77,960.00	0.279
Academic stress	9.0 (8.0–10.0)	9.0 (7.0–10.0)	77,104.00	0.413
Social stress	9.0 (8.0–10.0)	9.0 (7.0–10.0)	78,240.50	0.237
Economic stress	7.0 (6.0–8.0)	7.0 (6.0–8.0)	74,193.00	0.913

* *p*-value < 0.05, ** *p*-value < 0.01.

**Table 2:** Multifactor binary logistic regression (stepwise adjustment model).

Variable	Model 1			Model 2			Model 3			Model 4		
	OR	95% CI	*p*-Value	OR	95% CI	*p-*Value	OR	95% CI	*p*-Value	OR	95% CI	*p*-Value
Screentime	1.13	1.04–1.23	0.004 **	1.14	1.05–1.25	0.002 **	1.14	1.05–1.24	0.003 **	1.13	1.04–1.24	0.006 **
Sugary beverage intake	1.54	1.02–2.30	0.038 *	1.58	1.05–2.38	0.03 *	1.57	1.04–2.38	0.032 *	1.48	0.97–2.28	0.072
Outdoor activity	0.69	0.47–1.01	0.057	0.64	0.43–0.95	0.027 *	0.64	0.43–0.94	0.024 *	0.63	0.42–0.95	0.029 *
Fried food intake	1.28	0.86–1.91	0.225	1.27	0.85–1.91	0.246	1.24	0.83–1.87	0.297	1.23	0.80–1.88	0.35
Vegetable intake	0.6	0.42–0.87	0.007 **	0.61	0.42–0.88	0.009 **	0.6	0.41–0.87	0.008 **	0.58	0.39–0.85	0.006 **
Fruit intake	0.69	0.47–1.00	0.048 *	0.69	0.47–1.00	0.052	0.67	0.46–0.98	0.041 *	0.67	0.45–0.99	0.045 *
Smoking	1.76	1.03–2.99	0.038 *	1.86	1.09–3.19	0.023 *	1.87	1.09–3.22	0.024 *	1.9	1.08–3.33	0.025 *
Alcohol consumption	1.36	0.94–1.97	0.100	1.33	0.91–1.94	0.138	1.33	0.91–1.94	0.138	1.27	0.86–1.88	0.232
Spicy preference	1.64	1.16–2.31	0.005 **	1.7	1.18–2.45	0.004 **	1.7	1.18–2.45	0.005 **	1.68	1.15–2.46	0.007 **
Use of skincare products	1.64	1.21–2.23	0.001 **	2.07	1.38–3.11	0.000 **	2.09	1.39–3.15	0.000 **	2.02	1.33–3.07	0.001 *
Age				1.05	0.77–1.43	0.748	1.05	0.77–1.42	0.773	1.03	0.75–1.41	0.854
Gender				0.7	0.46–1.05	0.084	0.69	0.46–1.05	0.081	0.72	0.47–1.10	0.134
Place of origin				0.9	0.62–1.29	0.554	0.9	0.62–1.29	0.552	0.89	0.61–1.30	0.558
Grade 2				0.92	0.53–1.60	0.763	0.95	0.54–1.67	0.864	0.91	0.51–1.63	0.751
Grade 3				0.99	0.43–2.32	0.986	1.00	0.43–2.34	0.994	1.08	0.45–2.61	0.860
Grade 4				0.82	0.21–3.25	0.774	0.85	0.21–3.39	0.813	0.86	0.21–3.57	0.832
Grade 5				1.17	0.22–6.34	0.857	1.21	0.22–6.63	0.826	1.23	0.21–7.07	0.818
Major—History & Philosophy				0.70	0.32–1.52	0.371	0.71	0.32–1.53	0.379	0.65	0.29–1.44	0.291
Major—Education				1.03	0.51–2.08	0.945	0.99	0.49–2.00	0.97	0.89	0.43–1.85	0.761
Major—Science & Engineering				1.40	0.68–2.86	0.361	1.38	0.67–2.84	0.375	1.2	0.57–2.53	0.622
Major—Economic & Management				0.99	0.48–2.04	0.977	0.97	0.47–2.00	0.929	0.81	0.38–1.71	0.577
Sleep quality							1.11	1.03–1.20	0.008 **	1.1	1.02–1.20	0.019 *
Academic stress										1.37	1.24–1.51	0.000 **
Social stress										1.06	0.97–1.17	0.172
Financial stress										1.15	1.00–1.33	0.052
Auc	0.652			0.665			0.674			0.720		

Note: the blank cells indicate that the corresponding variable was not included in that stepwise regression model. * *p*-value < 0.05, ** *p*-value < 0.01.

**Table 3:** Subgroup analysis.

Category	Subgroup	OR	95% CI Lower	95% CI Upper	*p*-Value Interact
Sex	Male (Ref)	1.164	1.076	1.258	0.350
Sex	Female	1.232	1.128	1.345	
Grade Group	Low Grade (Fresh/Soph)	1.184	1.093	1.282	0.690
Grade Group	High Grade (Junior/Senior/5th)	1.210	1.111	1.318	
Major Group	Non-Medical	1.180	1.112	1.252	0.103
Major Group	Medical (Ref)	1.566	1.101	2.228	
Outdoor Activity	No	1.128	0.993	1.281	0.317
Outdoor Activity	Yes	1.209	1.132	1.291	
Origin	Rural (Ref)	1.172	1.086	1.264	0.489
Origin	Urban	1.222	1.116	1.337	

**Table 4:** Interaction test.

Interaction Term	Odds Ratio	95% CI Lower	95% CI Upper	*p*-Value
Gender × Stress	1.079	0.950	1.225	0.244
Sleep Quality × Stress	1.023	0.990	1.057	0.179
Gender × Sleep Quality	1.057	0.894	1.249	0.517
Stress × Sugary Drink Intake	1.131	0.964	1.327	0.131

**Table 5:** Sensitivity analysis.

Variable	Coef	OR	95% CI Lower	95% CI Upper	*p*-Value	E-Value
Total stress	0.171	1.187	1.118	1.260	0.000 **	1.658
Sleep quality	0.104	1.109	1.025	1.201	0.010 *	1.458
Screen time	0.101	1.107	1.015	1.206	0.021 *	1.450
Sugary beverage intake	0.445	1.561	1.052	2.315	0.027 *	2.497
Outdoor activity	−0.391	0.676	0.459	0.996	0.048 *	2.321
Spicy preference	0.553	1.739	1.203	2.513	0.003 **	2.873
Smoking	0.593	1.809	1.057	3.097	0.031 *	3.019
Use of skincare products	0.695	2.004	1.333	3.012	0.001 **	3.422

* *p*-value < 0.05, ** *p*-value < 0.01.

**Table 6:** Robustness test.

Variable	Original OR	Original *p*	NonMed OR	NonMed *p*	NoOutlier OR	NoOutlier_*p*	Selected_by_LASSO
Age	1.049	0.374	1.034	0.540	1.033	0.546	FALSE
Gender	0.737	0.143	0.730	0.144	0.744	0.158	TRUE
Total Stress	1.187	0.000	1.175	0.000	1.198	0.000	FALSE
Sleep Quality	1.109	0.010	1.105	0.016	1.119	0.007	TRUE
Screen Time	1.107	0.021	1.116	0.015	1.090	0.054	TRUE
Sugary beverage intake	1.561	0.027	1.470	0.063	1.545	0.031	TRUE
Outdoor activity	0.676	0.048	0.643	0.028	0.690	0.062	TRUE
Spicy preference	1.739	0.003	1.756	0.003	1.706	0.005	TRUE
Smoking	1.809	0.031	1.726	0.055	1.813	0.030	TRUE
Use of skincare products	2.004	0.001	1.935	0.002	2.026	0.001	TRUE

## Data Availability

The raw data supporting the conclusions of this article will be made available by the authors on request.

## Abbreviations

AUC	Area Under the Curve
C-CSI	Chinese College Student Stress Inventory
CI	Confidence Interval
HPA	Hypothalamic–Pituitary–Adrenal
IL	Interleukin
OR	Odds Ratio
PSQI	Pittsburgh Sleep Quality Index
ROC	Receiver Operating Characteristic
TEWL	Transepidermal Water Loss
TNF-α	Tumor Necrosis Factor-α
